# Solid-state optical properties of self-assembling amyloid-like peptides with different charged states at the terminal ends

**DOI:** 10.1038/s41598-021-04394-2

**Published:** 2022-01-14

**Authors:** Chiara Schiattarella, Carlo Diaferia, Enrico Gallo, Bartolomeo Della Ventura, Giancarlo Morelli, Luigi Vitagliano, Raffaele Velotta, Antonella Accardo

**Affiliations:** 1 Institute of Applied Sciences and Intelligent Systems, CNR, Via P. Castellino 111, 80131 Naples, Italy; 2 Department of Pharmacy and Research Centre on Bioactive Peptides (CIRPeB), University of Naples “Federico II”, Via Mezzocannone 16, 80134 Naples, Italy; 3 IRCCS Synlab SDN, Via Gianturco 113, 80143 Naples, Italy; 4 Department of Physics “Ettore Pancini”, University of Naples “Federico II”, Via Cintia 26, 80125 Naples, Italy; 5 Institute of Biostructures and Bioimaging (IBB), CNR, Via Mezzocannone 16, 80134 Naples, Italy

**Keywords:** Chemistry, Materials science, Nanoscience and technology, Optics and photonics, Physics

## Abstract

The self-assembling of small peptides not only leads to the formation of intriguing nanoarchitectures, but also generates materials with unexpected functional properties. Oligopeptides can form amyloid-like cross-β assemblies that are able to emit intrinsic photoluminescence (PL), over the whole near-UV/visible range, whose origin is still largely debated. As proton transfer between the peptide chain termini within the assembly is one of the invoked interpretations of this phenomenon, we here evaluated the solid state PL properties of a series of self-assembled hexaphenylalanine peptides characterized by a different terminal charge state. Overall, our data indicate that the charge state of these peptides has a marginal role in the PL emission as all systems exhibit very similar multicolour PL associated with a violation of the Kasha’s rule. On the other hand, charged/uncharged ends occasionally produce differences in the quantum yields. The generality of these observations has been proven by extending these analyses to the Aβ_16–21_ peptide. Collectively, the present findings provide useful information for deciphering the code that links the spectroscopic properties of these assemblies to their structural/electronic features.

## Introduction

**Figure 1:**
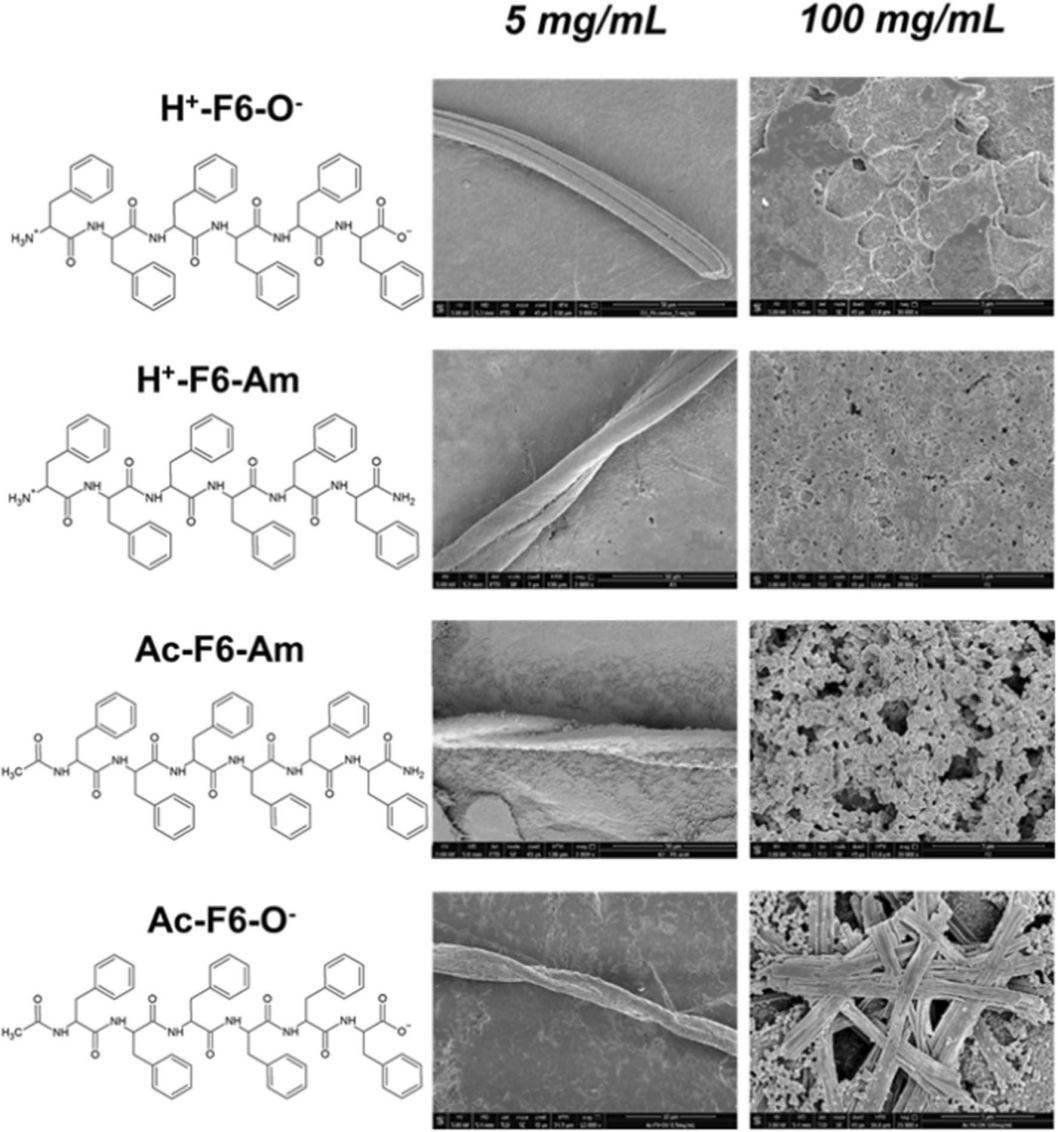
Schematic representation of F6-peptide variants; selected SEM micrographs for peptides at 5.0 and 100 mg/mL (50 mg/mL for H^+^-F6-O^−^); the scale bars are 50 μm and 5 μm, respectively.

Peptide-based nanostructures are progressively gaining research interest as they represent innovative tools in different fields including biomedicine and biotechnology^1–6^. Depending on their primary sequence, peptides are able to self-assemble into a variety of supramolecular structures characterized by different architectures. These structures are directly affected by the secondary organization they can adopt (α-helix or β-sheet)^7^. Notably, the interest for small synthetic peptides goes well beyond their structural ability to self-assemble, as they also present intriguing functional properties including piezoelectricity^8,9^, magnetism^10^ and photoluminescence (PL)^11,12^. This latter property consists in an unexpected optical emission exhibited by both natural and synthetic amyloid-like structures in the range 400 nm < λ_em_ < 560 nm upon their excitation between 380 and 460 nm. The first study describing this unusual phenomenon was reported in protein crystals and large polypeptides by Shukla et al.^13^. Later, very similar intrinsic blue/green photoluminescence was observed in protein fibrils or nanostructures originated by self-assembly of short peptide sequences^14–16^. Very recent studies have further expanded these observations by showing that amyloid-like peptides and proteins are able to emit fluorescence in the near infrared region upon excitation at 650–700 nm^17,18^. This strong experimental evidence is somehow in contrast with the classical frame of fluorescence properties of proteins/peptides, which states that only the three aromatic amino acids (Phe, Tyr and Trp) are able to emit fluorescence in the far UV region^19^. The unusual PL properties of amyloid-like systems have been related to the β-sheet organization that characterizes these assemblies. At the state of art, several other hypotheses have been advanced to explain the origin of this PL associated with β-sheets. Nevertheless, the physicochemical basis of this phenomenon has not been identified yet, although several proposals have been reported and discussed in literature^20^. The most accredited hypotheses include: (I) electron delocalization through intra/intermolecular hydrogen bonds^21^; (II) quantum confinement effects^22^; (III) charge transport through the H bond networks^23,24^; (IV) carbonyl-based autofluorescence^25^; (V) nuclear quantum effect^26^; (VI) extensive delocalization of peptide bond electrons in the β-structure^27^. In 2016, Pinotsi et al*.* reported an interesting study in which PL emission from amyloid-like fibrils was correlated to the proton transfer between the C- and the N- termini of two adjacent strands in the β-sheet structure^23,24^. Despite an alternative explanation, involving the deformation of the peptide bond planarity in the assembly, has been successively reported^27^, the contribution of the proton transfer in the origin of the PL properties has not been further highlighted from the experimental point of view^27^. In this framework, we have recently undertaken extensive structural and spectroscopic investigations on analogous amyloidogenic systems constituted by self-assembled phenylalanine-based homopeptides, specifically in form of hexa-Phe (F6). Due to their hydrophobic nature, they were frequently conjugated to PEG (polyethylene glycol) moieties of different length to increase their solubility and to study their properties in solution (PEG8-F6, PEG12-F6, PEG18-F6 and PEG24-F6)^21,28,29^. Moreover, by studying a series of F6 variants endowed with either free or capped N-/C- termini we have also investigated the effects that charged ends play on the aggregation mechanism and on the structural properties of the final fibers^30^. Since the charged state of the peptide end crucially affects the probability of proton transfer between the termini, we here perform a comparative analysis of the photoemissive properties of four peptide analogues having the same peptide sequence (F6) and different charged states of their termini (see Fig. 1). Together with the zwitterionic peptide H^+^-F6-O^−^, which has charged termini, we characterized the fully capped peptide Ac-F6-Am (in which the C- and the N-termini are amidated and acetylated, respectively), and the two intermediate variants in which the termini are alternatively capped or uncapped, Ac-F6-O^−^ and H^+^-F6-Am. Collectively, these four variants provide all possible combinations of charged/uncharged states on the F6 peptide, thus allowing an insightful comparison of the dependence of fluorescence behaviour as function of the terminal state. Indeed, our previous structural/morphological characterization pointed out the tendency of these peptidic variants to self-assemble in amyloid-like structures, with an organization in β-sheet nanostructures with an antiparallel orientation of the β-strands in the cross-β framework, regardless their terminal charge^30^. The structural resemblance of the supramolecular organization of these nanostructures indicates that potential differences in the PL properties among different peptides can only be ascribed to their different chemical properties. The solid-state spectroscopic characterization of these compounds reported in the current work unravel some interesting features such as (1) the independence of the PL emission from the charge states of the peptide termini, (2) a multicolour PL emission, and (3) the general violation of the Kasha’s rule^31^. We also demonstrate that F6 films are stable against photodegradation and bleaching, an essential feature required for nanophotonic applications. The generality of the findings obtained for the F6 peptides are proven by studying the completely charged and uncharged variants of the Aβ_16–21_ peptide (H^+^-Aβ_16–21_-O^−^ and Ac-Aβ_16–21_-Am), which self-assemble in amyloid-like structures analogously to F6 variants.

## Results and discussion

### F6 film preparation and scanning electron microscopy (SEM)

F6 peptides were synthetized by solid phase peptide synthesis using Wang or Rink amide resin, which allow to achieve carboxylated and amidated variants, respectively^30^. The capping of one or both the termini allows to obtain peptides with different charge states. Due to their aromatic and hydrophobic nature, all peptides are scarcely soluble in water (~ 0.5 mg/mL). On the contrary, they are highly soluble (up to 100 mg/mL) in HFIP (1,1,1,3,3,3-hexafluoro-2-propanol), generally used as solvent/co-solvent for highly hydrophobic Phe-containing peptides^32,33^. Only the zwitterionic form, H^+^-F6-O^−^, showed a reduced solubility in HFIP (50 mg/mL). The effective capability of these peptides to give zwitterionic form in HFIP has been assessed by the Safranine T qualitative assay. Safranine T (reported also as Safranine O or basic red 2) is cationic azonium compounds of symmetrical 2,8-dimethyl-3,7-diaminophenazine able to interact with negative charged via electrostatic interactions^34^. This assay was carried out on samples in their solid state. The selected peptides were the completely protected peptide Ac-F6-Am—used as negative control—and, on the unprotected one, H^+^-F6-O^−^ used as positive control. Peptides were drop-casted on a slide glass from 5.0 mg/mL HFIP stock solutions and air-dried at room temperature. The resulting films were stained using a pure ethanol Safranine T solution at 0.30 mg/mL and imaged by optical microscopy under bright and cross polarized light. From the inspection of images in Fig. S1, an effective interaction between the peptide film and the dye only for H^+^-F6-O^−^ appears, thus confirming the existence of charges on the peptide under these experimental conditions. PL properties of these F6-peptides were investigated only for the samples at solid state, prepared by drop-casting peptide solutions at two different concentrations: 5 mg/mL and 100 mg/mL (50 mg/mL for H^+^-F6-O^−^). Prior to PL characterization, we evaluated the morphology of the peptide nanostructures at both the concentrations by Scanning Electron Microscopy (SEM). SEM micrographs in Fig. 1 allow to observe a dependence of the morphology from the concentration. At low concentration, the four variants assemble in twisted structures with a length and a thickness between 300 and 1000 μm and 5–20 μm, respectively. Instead, at high concentration, SEM micrographs show a sort of film formed by very short fibrillary structures aligned along all the directions, thus suggesting a lower degree of order with respect to samples at 5 mg/mL. This decrease of order is probably due to the high volatility and rapid evaporation of the organic solvent. Although our SEM analysis are only qualitative, there seems to be a somewhat higher degree of order in the two acetylated peptides with respect to the not acetylated ones. The optoelectronic properties of the air-dried F6 samples were preliminarily studied via fluorescence microscopy. The images of fibers and films in the dark field, in the blue and green regions are reported in Figs. S2 and 2, respectively. In clear accordance with the SEM characterization, the images showed µm-long fibrillary structures and films formed by ~ 100 µm-wide cracked plaque structures.

**Figure 2:**
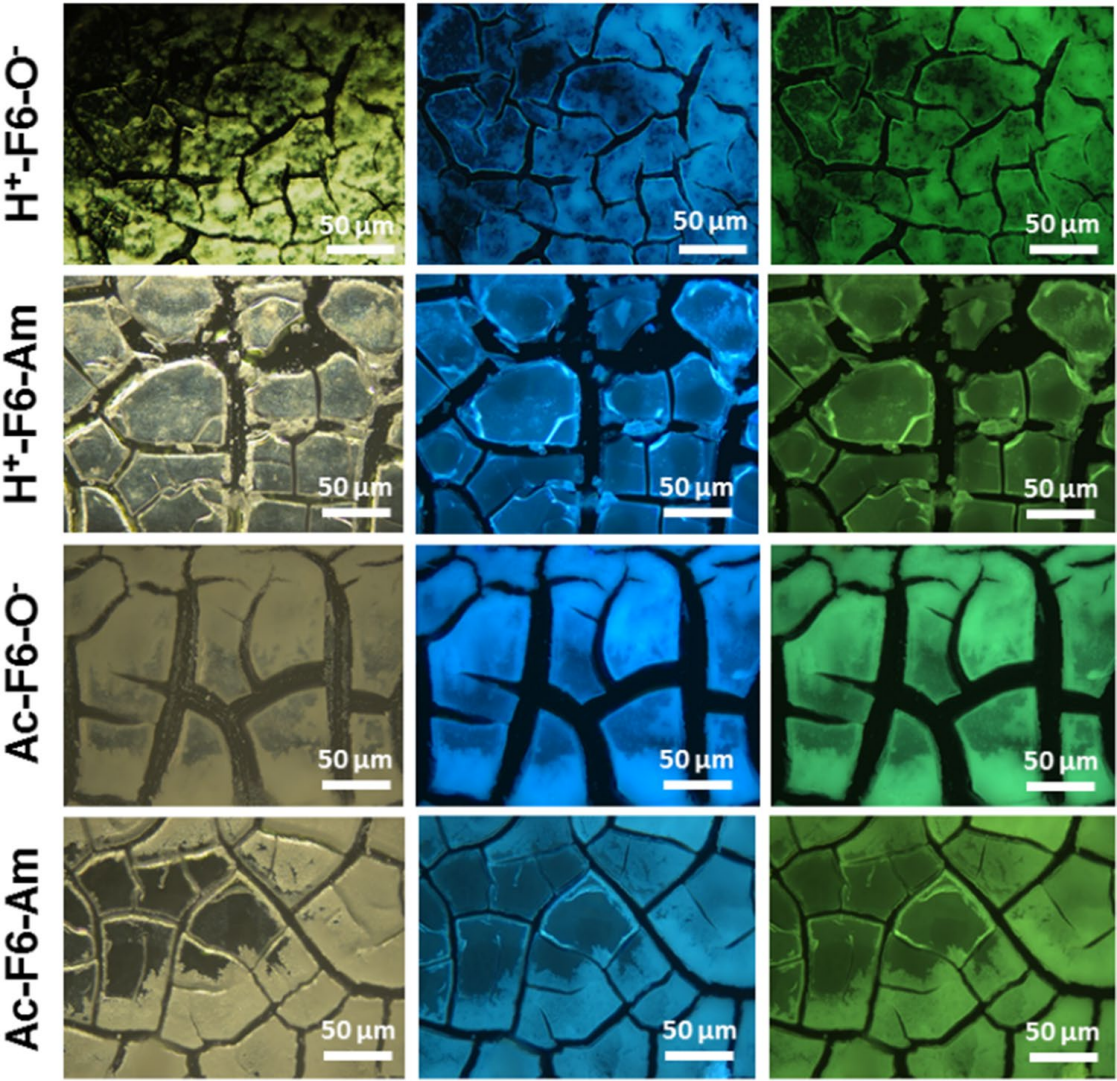
Optical microscopy images of all the F6 variants films, deposited on clean coverslip glass and air-dried at room temperature. From left to right: dark field images and fluorescence images excited in the spectral regions of DAPI (λ_exc_ = 359 nm, λ_em_ = 461 nm) and GFP (λ_exc_ = 488 nm, λ_em_ = 507 nm).

**Figure 3:**
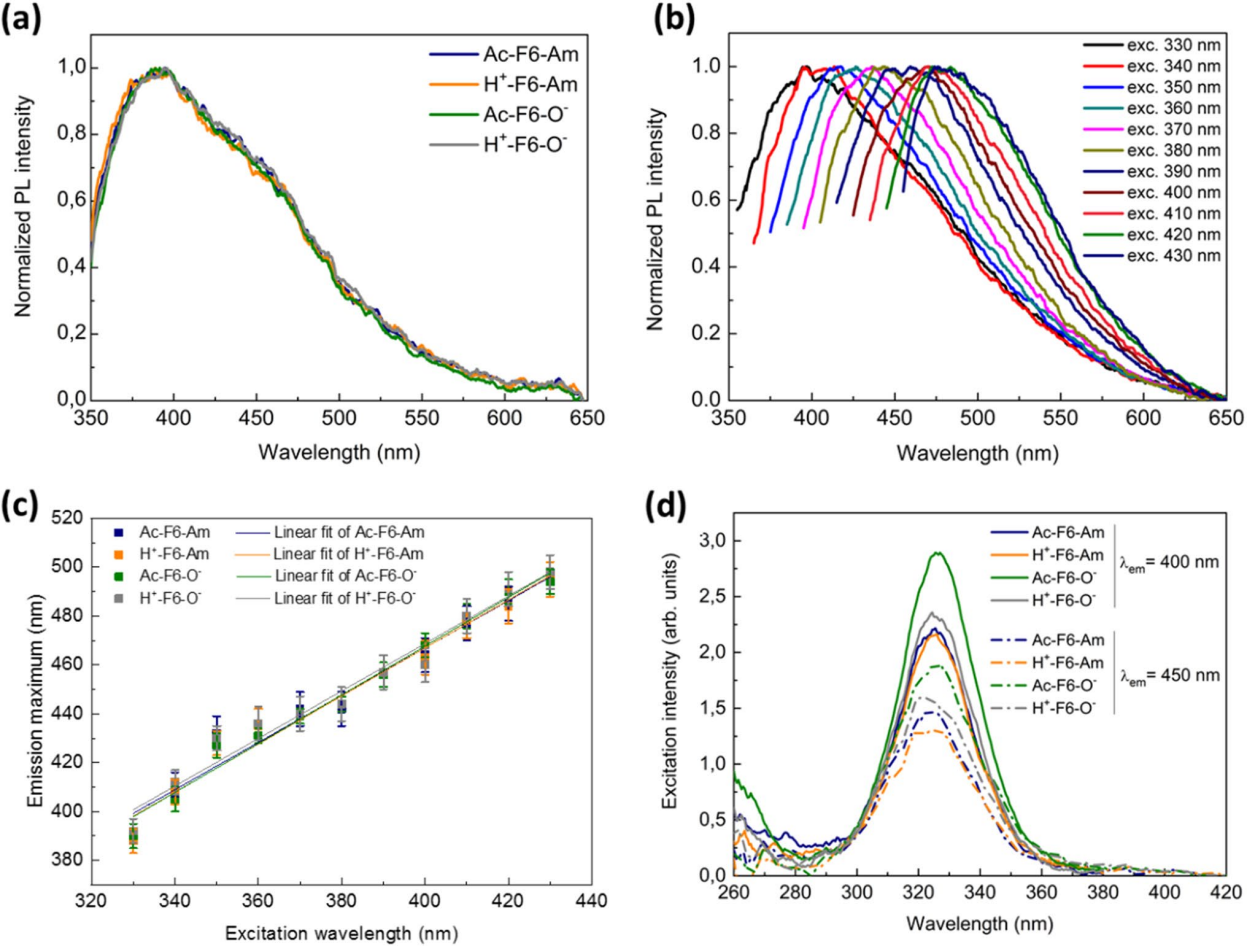
(**a**) Normalized PL spectra of the four F6 variants at λ_exc_ = 330 nm. (**b**) Representative normalized fluorescence spectra of a peptide film (herein Ac-F6-Am is reported) versus the excitation wavelength in the range between 330 and 430 nm. Herein, the data were normalized with respect to the maximum of the emission spectrum and rescaled within the interval [0,1] in order to highlight the shift of the maxima position at increasing excitation wavelengths. (**c**) Plots of the maxima positions as function of the excitation wavelength of all peptides. Therein, the linear best-fit curves are also reported. (**d**) Excitation spectra of the F6 samples setting λ_em_ = 400 nm (solid lines) and λ_em_ = 450 nm (dash-dotted lines). The concentration of the samples was fixed at 5 mg/mL.

Successively, the optical properties of the F6 nanostructures were extensively investigated from a quantitative point of view: from the spectral emissive viewpoint, all peptides exhibit a characteristic PL in the visible spectrum, having two main emission bands evidenced at ~ 400 and ~ 450 nm (Fig. 3a). This finding was independent of the concentration of the precipitating solution. This trace, not ascribable to the π-π stacking between the phenyl ring of Phe residues^28,35^, can rather be attributed to the presence of β sheet-rich nanostructures^15,22^.

A deeper investigation of this fluorescence emission clearly indicates that all four peptides present a highly similar multicolour emission profile as evidenced by the normalized PL spectra versus the excitation wavelength in the range from 330 to 430 nm (Figs. 3b, S3). This PL behaviour was previously reported for thermally treated triphenylalanine nanodots by Rosenman et al.^32^. Moreover, all F6 peptides show a fluorescence emission that is almost linearly dependent from the excitation wavelength in the whole investigated range (Figs. 3c, d and S3). Also, very recent outcomes have shown the occurring of this same effect in solid-state polyethylene glycol-derivatized F6 films^36^. The variation of the emission wavelength upon increasing the excitation wavelength exhibited by these peptides represents an interesting violation of the Kasha’s rule, according to which “the emitting level of a given multiplicity is the lowest excited level of that multiplicity”^31^. The violation of the rule implies the possibility that the photons can be emitted by a higher energy state thereby showing a dependence on the excitation wavelength; in particular, the shorter the excitation wavelength the shorter the emission wavelength. Indeed, a linear behaviour could be clearly evidenced for all the four F6 compounds, with slopes and intercepts fully compatible each other for all the compounds (Table 1). In order to gain further insights into this intricate process, we measured the excitation spectra corresponding to the main PL components (λ_em_ = 400 nm—continuous lines—and 450 nm—dash-dotted lines). As shown in Fig. 3d, the spectra exhibit a single well-defined peak, centred at ~ 325 nm for all four peptides, regardless the excitation wavelength. Optical transitions at such low energy in self-assembling β-rich polypeptides/proteins have been initially correlated to a putative long-range charge delocalization along their backbone due to hydrogen bonding occurring between peptide units^13^. This hypothesis has been supported by the evidence that hydrophobic conditions (low humidity and pressure) occurring in amyloid fibrillary systems hamper both PL and charge transport^16^. As a whole, the present data clearly demonstrate that this excitation-dependent emissive behaviour in the visible range as well as the low-energy excitation peak are invariant for all samples regardless their terminal charge. Therefore, in the examined systems, the photochemical explanation of this phenomenon seems not to be attributable to proton transfer or other mechanisms involving the termini of the peptide like few studies suggest^23,24^. Nevertheless, the results are coherent with very recent theoretical findings reported in the literature^27^, according to which the emission is promoted to the suppression of non-radiative paths due to the decreasing of the excitation energy and stabilization of n → π* transitions (*i.e.*, electrons belonging to non-bonding pair jumping up to an antibonding π* orbital). This is shown to be the consequence of a deplanarization of amide groups, which may lead to the peculiar supramolecular organization of the examined compounds^27^. Moreover, other studies demonstrate that PL from H-bonds rich, non-aromatic systems arises consequently to interactions between amide groups: the abundancy of hydrogen bonding was demonstrated to bring these functionalities into close proximity^37^. Indeed, the results reported in the current work are in good agreement with these proposed models as they do not invoke any role for the charged ends.

**Table 1:** Summary of the estimated quantities of the linear fits reported in Fig. 3c together with their relevant statistic parameters.

Sample	Slope	Intercept	Reduced χ^2^	Adj. R^2^
Ac-F6-Am	0.97 ± 0.06	80 ± 23	1.08	0.962
H^+^-F6-Am	0.98 ± 0.06	74 ± 23	1.14	0.962
Ac-F6-O^−^	1.00 ± 0.05	69 ± 18	1.125	0.976
H^+^-F6-O^−^	0.97 ± 0.06	80 ± 23	1.135	0.962

The photoemissive behaviour of the F6 films is slightly modified upon increasing the peptide concentrations in the mother solution since another excitation band centred at around ~ 370 nm arises differently, in addition to the component at 325 nm. In this case a small but significant dependence on the terminal charge states is observed as the peak is observed only for the two peptides with the amidated C-terminal end (Fig. S4a). This experimental evidence allows to conclude that, when the peptide is prepared from highly concentrated solutions (100 mg/mL), the charge state of the terminal ends also plays a role in the fluorescence emission, attributed to Phe π–π stacking and induced by exciting the samples at 257 nm (Fig. S4b). Indeed, in these conditions, an increased emission of the peptides with the C-terminal capped ends compared to those with a charged –COO^−^ moiety is observed. This aggregative phenomenon arises at high concentrations of deposited material and it can still be contextualized in terms of “preferential direction” in the self-assembling. It is reasonable to conclude that the main contribution to the optical response of the F6 films, in the higher concentration regime, comes from the stacking of the huge number of aromatic residues. This analysis is also supported by the SEM images above discussed for the samples at high concentrations. Finally, measurements of fluorescence quantum yield (PLQY) at the two excitation wavelengths of 325 and 370 nm (Table 2) provide further insights into the PL emission of these systems. At 325 nm an increase of the average PLQY from ~ 5(1) to ~ 10(2)% is evidenced when going from 5 to 100 mg/mL. No specific effect of the charged/uncharged ends is observed for this PL increase. Although it is important to point out that PLQY only provides information about the integrated photoemissive behaviour of the samples and not about the exact origin and attribution of the spectral features of the samples. This finding points out that the concentration of the peptide in the stock solution has an impact on the resulting structure of the solid and on the related PL properties. The inspection of the PLQY obtained upon excitation at 370 nm, which has only been evaluated in the samples at higher concentrations, indicates some role of the charge state of the terminal as C-terminal capped variants present quantum yields higher than those exhibited by peptide containing –COO^−^ groups.

**Table 2:** Summary of the measured values of PLQY of all F6 samples at the different concentrations.

Sample	F6 concentration (mg/mL)	QY at 325 nm (%)	QY at 370 nm (%)
Ac-F6-Am	5	5.8 ± 0.3	–
	100	12.1 ± 0.6	6.3 ± 0.3
H^+^-F6-Am	5	3.6 ± 0.2	–
	100	11.3 ± 0.6	9.1 ± 0.5
Ac-F6-O^−^	5	5.3 ± 0.3	–
	100	10.0 ± 0.5	4.4 ± 0.2
H^+^-F6-O^−^	5	4.4 ± 0.2	–
	50	12.7 ± 0.6	2.5 ± 0.1

### Photobleaching study

**Figure 4:**
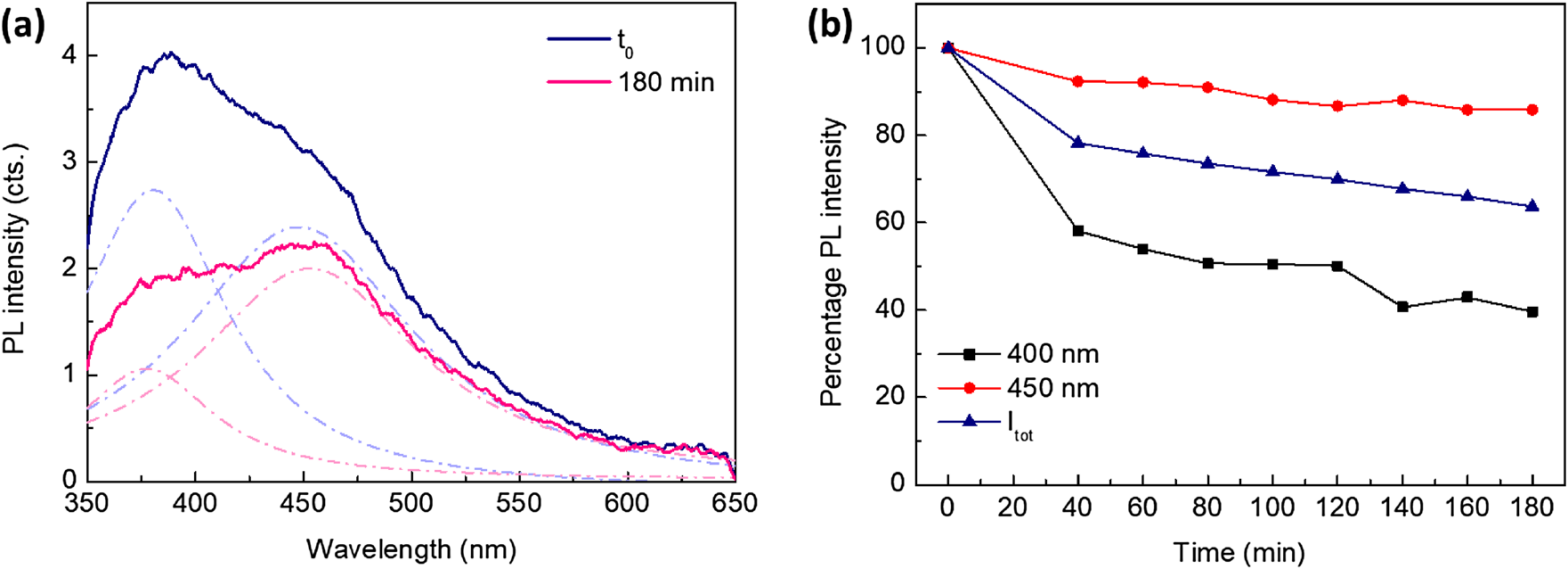
(**a**) PL spectrum of Ac-F6-Am (and its deconvolved components—dash-dotted lines) at t = 0 and after 180 min continuous exposure to 330 nm light. (**b**) Bleaching kinetic behavior of the 400 nm (black squares) and 450 nm PL components (red circles). The kinetics of the whole integrated signal as a function of the exposure time (blue triangles) is reported as well.

Having shed light upon the intriguing spectroscopic properties of the nanostructures formed by F6 peptides, we evaluated their robustness against photodegradation and bleaching, which represent essential features to pursue any nanophotonic application. For this experiment, fluorescence spectra were collected and monitored after continuous exposure to the 330 nm excitation line, keeping the sample mounted in the integrating sphere and the excitation shutter open, for up to 180 min at an incident power of ~ 820 μW. Assuming the interchangeability of the four variants in view of the previous results, the measurements were solely carried out on the Ac-F6-Am peptide film at 100 mg/mL concentration. After 3 h continuous exposure to UV light, a 64% retaining of the integrated PL intensity of the sample could be evidenced, revealing a remarkable bleaching resistance (Fig. 4a). A rough estimation of the bleach rate, performing a single-exponential decay fit, led to a value of 7.2 (1.1) h, which is much higher than that of any conventional protein dye (whose characteristic range is ~ 10^1^–10^2^ s)^38^. Furthermore, the two deconvolved components of the PL spectrum (peaked at ~ 400 and ~ 450 nm) interestingly exhibit a different bleaching kinetics, highlighting the different origin of their photoemissive mechanisms (Fig. 4b). Moreover, no recovery was evidenced after 40 min in dark (data not shown).

### Photoemissive properties of Aβ_16–21_ peptides

**Figure 5:**
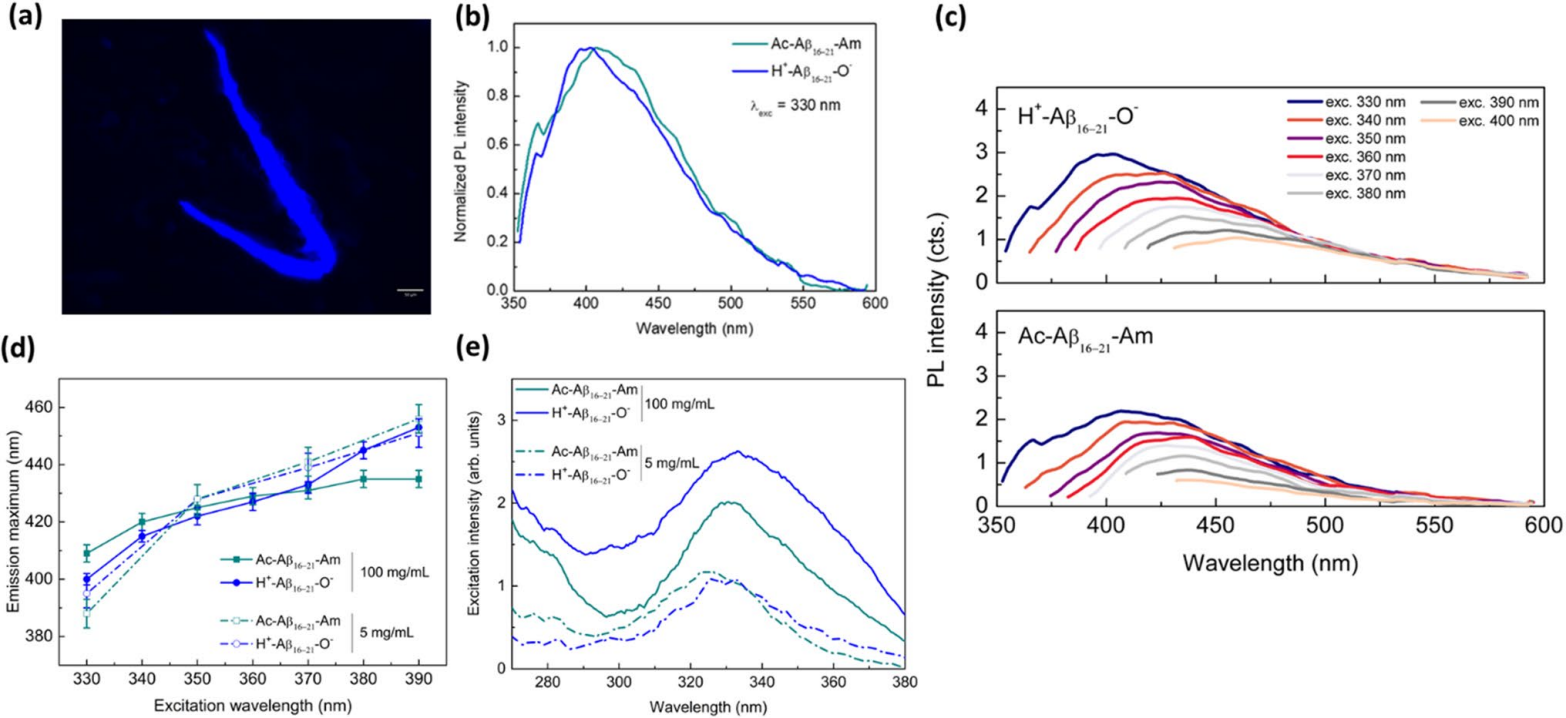
(**a**) PL microscopy image of Ac-Aβ_16–21_-Am drop-casted on a clean coverslip glass in the DAPI region. The scale bar is 50 μm. (**b**) Representative normalized PL spectra of the two characterized Aβ_16–21_ peptides at λ_exc_ = 330 nm. Analogously to Fig. 3, the data were normalized with respect to the maximum of the emission spectrum and rescaled within the interval [0,1] in order to highlight the overlap of the spectral features of the Aβ_16–21_ samples at fixed excitation wavelength. (**c**) PL spectra of Aβ-peptide films versus the excitation wavelength in the range between 330 and 400 nm. (**d**) Plots of the PL maxima versus excitation wavelength. Herein, the lines depicted represent a guide to the eye. (**e**) Excitation spectra of the same samples at different concentrations. For both (**d**) and (**e**), continuous lines: 100 mg/mL; dash-dotted lines: 5 mg/mL.

In order to demonstrate that the PL results collected for F6 nanostructures can be generalized to other classes of β-sheet-assembling peptides, we investigated another amyloidogenic system. Specifically, for this study we choose the sequence 16–21 “extracted” from the Aβ_1–42_ peptide (Aβ_16–21_), in its fully neutral (Ac- Aβ_16–21_-Am) and zwitterionic state (H^+^-Aβ_16–21_-O^−^) (see Fig. S5a). Analogously to their parental peptide Ac-Aβ_16–20_-Am^39^, the structural characterization on Aβ_16–21_ variants (SEM and ThT assay) confirmed their capability to self-organize in β-sheet-rich nanostructures (see Fig. S5b and S5c). The PL characterization of Aβ-peptide films at 5 and 100 mg/mL was carried out using the same setting chosen for the F6 samples. Due to the increased solubility in water of Aβ variants respect to F6 ones, samples at 5 mg/mL were prepared from their aqueous solutions. Analogously to the F6-peptides, PL microscopy images of Aβ (drop-casted from a solution at 5 mg/mL in H_2_O) show emissive fibrils in the blue spectral region (Fig. 5a). From the spectral viewpoint, both the Aβ variants show an emission peak around 400 nm (Fig. 5b), a multicolour PL emission profile in the range 330–400 nm (Fig. 5c) and a non-trivial dependence of the emission maximum on the excitation wavelength (Fig. 5d). Moreover, both the samples at 5 and at 100 mg/mL show a low-energy excitation peak at 325 nm (λ_em_ = 400 nm), which is compatible with what observed in F6 samples at the same concentration (Fig. 5e). The measured PLQYs at 100 mg/mL were (4.5 ± 0.2)% and (3.00 ± 0.15)% for H^+^-Aβ_16–21_-O^−^ and Ac-Aβ_16–21_-Am, respectively. Such values are comparable to those measured for the F6 variants in the same experimental conditions, as this peculiar emission turned out to exhibit a quite high efficiency. Altogether, such analyses support and are coherent with the assumption made for the F6, *i.e.*, that the excitation-dependent PL from an amyloidogenic system may be a direct consequence of a peculiar tridimensional-spatial disposition of the amide groups consequent to the β-sheet arrangement.

## Conclusions

The PL results collected on two amyloidogenic systems, F6 and Aβ_16–21_, allow to conclude that all the generated peptide films are able to emit multicolor PL profiles regardless their terminal charge state. The observed optical properties provide further support to the notion that proton transfer between the C- and the N-terminal of two adjacent strands in a β-sheet structure is not essential for generation of the intrinsic PL exhibited by amyloid-like systems.These results are compatible with the hypothesis that the emission is a consequence of the decrease of the excitation energy and stabilization of the peptide bond due to a deplanarization of amide groups^27^, although this explanation has to demonstrate its validity in interpreting the fluorescence exhibited by helical peptides^40^ as well as the its dependence from the physical state of the sample^36^.

The efficient excitation-dependent PL of the examined compounds was investigated and demonstrated to be retained even at higher concentrations where other aggregation-induced emissive phenomena occur, reaching PLQY values of the order of ~ 10%. The origin of this visible PL trace seems to be a direct consequence of their peculiar 3D-spatial disposition at the very first-stage supramolecular level.

## Materials and methods

Protected N^α^-Fmoc-Phe-OH, preloaded Fmoc-Phe-Wang resin, Rink amide MBHA (4-methylbenzhydrylamine) and all reagents for coupling reactions, commercially available from Calbiochem-Novabiochem (Laufelfingen, Switzerland), were used. All other chemicals materials, commercially available by Sigma-Aldrich (Milan, Italy), Fluka (Bucks, Switzerland) or LabScan (Stillorgan, Dublin, Ireland) were used as received unless otherwise stated.

### Peptide synthesis

F6-homosequences were synthesized according to the method previously described^30^. Ac-Aβ_16–21_-Am and H^+^-Aβ_16–21_-O^−^ were synthesized using the standard solid-phase 9-fluorenylmethoxycarbonyl (Fmoc) procedures. The Rink amide MBHA resin (substitution 0.65 mmol/g, 010 mmol) and the Wang resin preloaded with Ala (substitution 0.60 mmol/g, 010 mmol) were used as solid phase support. Wang resin provides C-terminus as carboxylic acid, meanwhile Rink amide resin releases peptide as amide at the C-terminus. The synthesis was carried out using a mixture of *N*,*N*-dimethylformamide/*N*-methyl-2-pyrrolidone (DMF/NMP, 1:1, v/v) as solvent phase. Before starting the peptide elongation, resins were swelled for 30 min in solvent medium. Fmoc deprotection was performed twice (each treatment for 10 min) using 30% (v/v) piperidine in DMF/NMP. The Fmoc-amino acid couplings were achieved by adding twofold molar excess of Fmoc-aa-OH, mixed with equimolar amounts of 1-hydroxybenzotriazole (HOBt), benzotriazol-1-yl-oxy-tris-pyrrolidino-phosphonium (PyBop) and fourfold molar excess of diisopropylethylamine (DIPEA). All couplings were performed twice for 40 min. *N*-terminus acetylation was performed twice (each treatment for 10 min) using a solution of pyridine/acetic anhydride (4/4.7 v/v) in DMF. Crude peptides were fully cleaved in acidic condition by TFA (trifluoroacetic acid)/H_2_O/ TIS (triisopropylsilane) (90/5/5 v/v/v) mixture at room temperature for 2 h. Then, peptides were precipitated with ice-cold ether and lyophilized. Purity and chemical identity of the synthetic products were assessed by analytical LC–MS analyses by using Finnigan Surveyor MSQ single quadrupole electrospray ionization (Finnigan/Thermo Electron Corporation San Jose, CA) using a column C18-Phenomenex eluted with an H_2_O/0.1% TFA (A) and CH_3_CN/0.1% TFA (B) from 5 to 70% over 20 min at 200 μL/min flow rate.

$$\begin{aligned} {\text{H}}^{ + } {\text{ - A}}\upbeta _{16 - 21} {\text{ - O}}^{ - } \;{\text{characterization:}}\;{\text{t}}_{{\text{R}}} & = 15.00\;\min ,\;{\text{MS}}\left( {{\text{ESI}} + } \right){:}\;{\text{m/z}}\;723.90\;{\text{calcd}}.\;{\text{for}}\;{\text{C}}_{38} {\text{H}}_{57} {\text{N}}_{7} {\text{O}}_{7} {:}\;\left[ {{\text{M}} + {\text{H}}^{ + } } \right] = 724.90 \\ {\text{Ac - A}}\upbeta _{16 - 21} {\text{ - Am}}\;{\text{characterization:}}\;{\text{t}}_{{\text{R}}} & = 15.46\;\min ,\;{\text{MS}}\left( {{\text{ESI}} + } \right){:}\;{\text{m/z}}\;764.95\;{\text{calcd}}.\;{\text{for}}\;{\text{C}}_{40} {\text{H}}_{60} {\text{N}}_{8} {\text{O}}_{7} {:}\;\left[ {{\text{M}} + {\text{H}}^{ + } } \right] = 764.46 \\ \end{aligned}$$

### Sample preparation

Ac-F6-O^−^, H^+^-F6-Am and Ac-F6-Am solutions were prepared dissolving peptide powders directly in 1,1,1,3,3,3-hexafluoro-2-propanol (HFIP) at 100 mg/mL. For H^+^-F6-O^−^, solution concentration was 50 mg/mL. Subsequently, these solutions were properly diluted with HFIP at the final required concentration. Ac-Aβ_16–21_-Am peptide solution was prepared directly dissolving lyophilized powder in double distilled water. The experimental concentration was assessed by UV–Vis spectroscopy carried out on UV–Vis Thermo Fisher Scientific Inc (Wilmington, Delaware USA) Nanodrop 2000c spectrophotometer using a 1.0 cm quartz cuvette (Hellma) and a molar absorptivity (ε257) of 390 M^−1^/cm. Then, these peptide solutions were drop-casted onto flat microscope glass slides and allowed to air dry at room temperature.

### Safranine T qualitative assay

Safranine T (Safranine O) was used as positively charged staining agent. 100 µL of peptide solutions of H^+^-F6-O^−^ and Ac-F6-Am were deposited from HFIP stocks at 5.0 mg/mL. After solvent evaporation at room temperature, peptide films were stained for 1 min with 50 µL of a 0.30 mg/mL Safranine T solution in anhydrous ethanol. After total dryness of the films, stained samples were inspected by optical microscope under bright-field illumination and between crossed polars by using a Nikon AZ100 microscope.

### Thioflavin T (ThT) assay

Aggregate formation of H^+^-Aβ_16–21_-O^−^ and Ac-Aβ_16–21_-Am was assessed by fluorescence spectroscopy using ThT. This dye associates rapidly with β-rich aggregates giving rise to an enhanced fluorescence emission at 482 nm^41^. Spectra of an aqueous solution of 25 µM ThT, before and after the addition of Ac-Aβ_16–21_-Am derivative (3.0 mg/mL), were recorded at room temperature. The spectrum of the peptide solution alone was also acquired as reference. Fluorescence measurements were recorded between 460 and 650 nm exciting the samples at 450 nm.

### Scanning electron microscopy (SEM)

Morphological analysis of the nanostructures was carried out using field emission scanning electron microscope (Nova NanoSem 450-FEI). Briefly, 10μL of sample solutions at 100.0 (50 mg/mL for H^+^-F6-O^−^) and 5.0 mg/mL were dropped off on aluminum stub using a graphite adhesive tape. A thin coat of Au and Pd was sputtered at a current of 20 mA for 120 s. The sputter coated samples were then introduced into the specimen chamber and the images were acquired at an accelerating voltage of 2–5 kV, spot 3, through the Everhart Thornley Detector (ETD) and the Through the Lens Detector (TLD).

### Fluorescence microscopy

10 μL of F6 and of Aβ_16–21_ peptide solutions were drop-casted on a clean coverslip glass, dried and imaged with fluorescence microscopy. Immunofluorescence images were taken with a Leica DM6 M fluorescence microscope equipped with DFC 7000 T camera, at excitation wavelengths 365 nm and 470 nm.

### Fluorescence measurements

The as-prepared samples were spectrally characterized using a Jasco FP-8300 spectrofluorometer equipped with an ILF-835 integrating sphere, which allowed us to evaluate the photoluminescence quantum yield (PLQY) with the absolute method thereby overcoming the issue of unavoidable differences in the relative emission intensities arising from poorly reproducible drop-casted peptide films. Fluorescence spectra of the four F6 films were acquired in a window up to 650 nm, probing the 330–430 nm excitation range at 10 nm steps. All acquisitions were averaged over five measurements after spectral correction.

### Photodegradation of peptide films

PL spectra were collected after continuous exposure to the 330 nm excitation line of the instrument, keeping the sample mounted in the integrating sphere and the excitation shutter open, for up to 180 min (at an incident power of ~ 820 μW).

## Supplementary Information

Supplementary Figures.
