# Supplementary material for: Solid-state optical properties of self-assembling amyloid-like peptides with different charged states at the terminal ends
Source: Sci Rep. 2022 Jan 14;12:759. doi: 10.1038/s41598-021-04394-2 (PMC8760239; doi:10.1038/s41598-021-04394-2)
Supplement: Supplementary file 1 — Supplementary Figures. [file 41598_2021_4394_MOESM1_ESM.pdf]

Supplementary Materials

Solid-state optical properties of self-assembling amyloid-like peptides with different charged states at the terminal ends

Chiara Schiattarella,[‡a] Carlo Diaferia[‡b,] Enrico Gallo,[c] Bartolomeo Della Ventura,[d] Giancarlo Morelli,[b] Luigi Vitagliano,[e] Raffaele Velotta*[d] and Antonella Accardo*[b]

[a]Institute of Applied Sciences and Intelligent Systems, CNR, Via P. Castellino 111, Naples, 80131, Italy.

[b]Department of Pharmacy and Research Centre on Bioactive Peptides (CIRPeB), University of Naples "Federico II", Via Mezzocannone 16, Naples, 80134, Italy.

Email: antonella.accardo@unina.it

[c]IRCCS SDN, Via Gianturco 113, Naples, 80143, Italy;

[d]Department of Physics "Ettore Pancini", University of Naples "Federico II", Via Cintia 26, Naples, 80125, Italy. Email:rvelotta@unina.it

[e]Institute of Biostructures and Bioimaging (IBB), CNR, Via Mezzocannone 16, 80134, Naples, Italy.

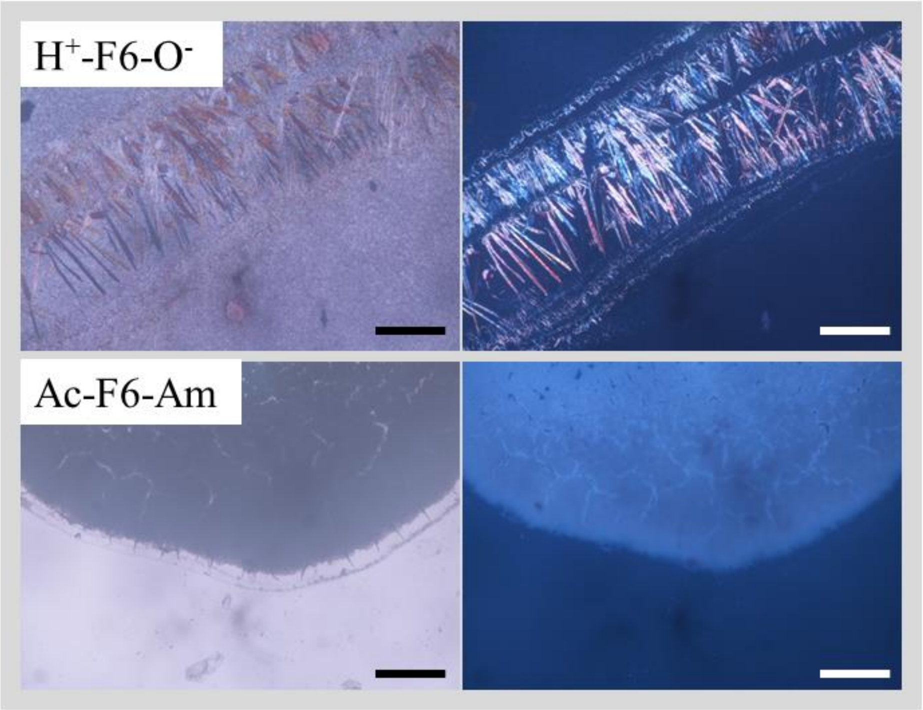

Figure S1: Qualitative Safranine T assay for H^{+}-F6-O^{-} and Ac-F6-Am. Peptide films were imaged under both brightfield (on the left) and polarized light (on the right). Scale bars are 100 µm.

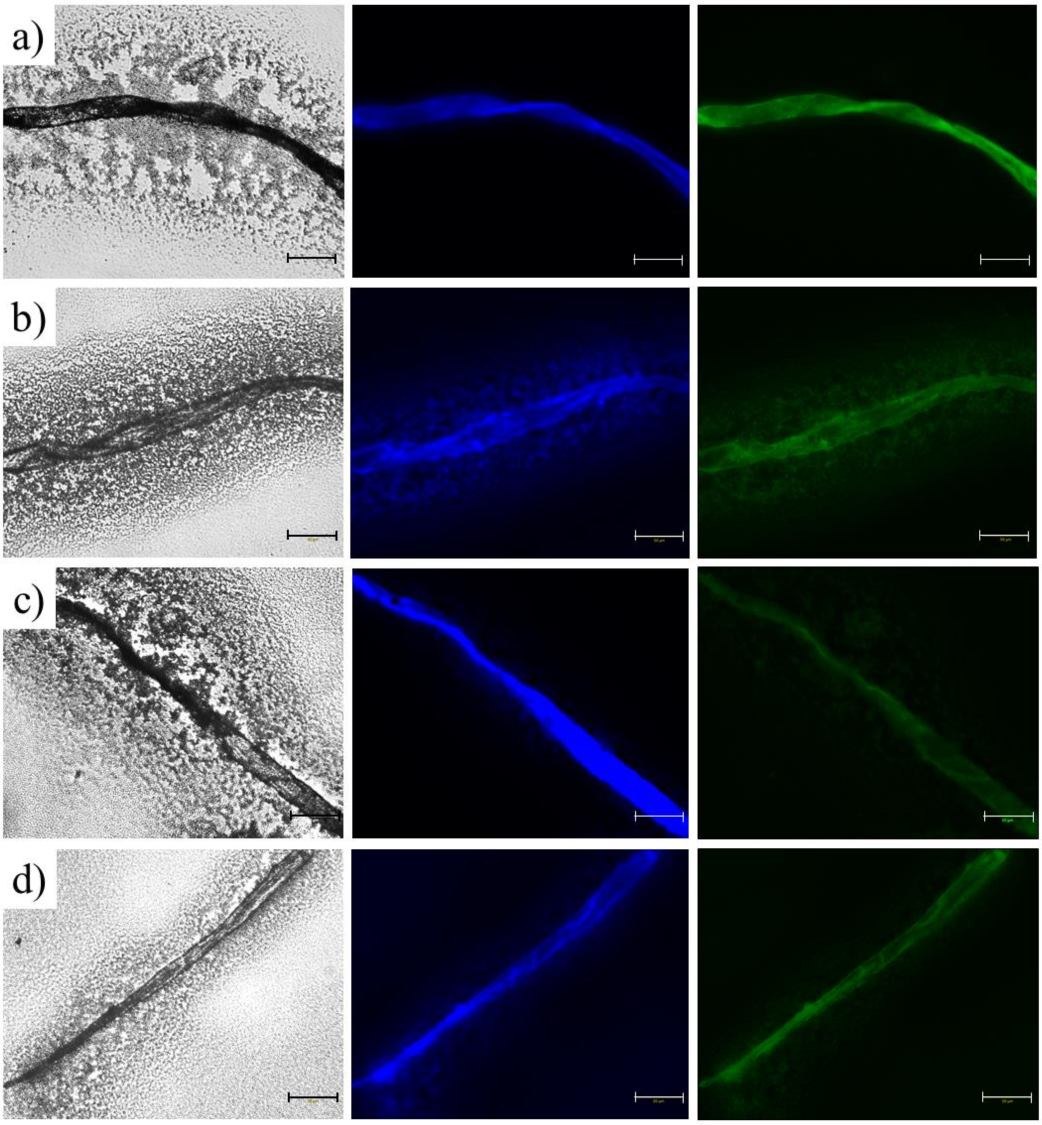

Figure S2: Fluorescence microscopy images of: a) H^+-F6-O^-, b) Ac-F6-O^-, c) H^+-F6-Amide and d) Ac-F6-Amide. All the samples are prepared by deposition of peptide solution (5.0 mg·mL^{-1}) in HFIP on a clean coverslip glass and slowly dried at room temperature. On the left, images in the bright field, in the center and on the right PL images in the DAPI (4',6-diamidino-2-phenylindole; λ_{exc} = 359 nm, λ_{em} = 461 nm) and GFP (Green Fluorescent protein λ_{exc} = 488 nm, λ_{em} = 507 nm) spectral regions. The scale bar = 50 μm.

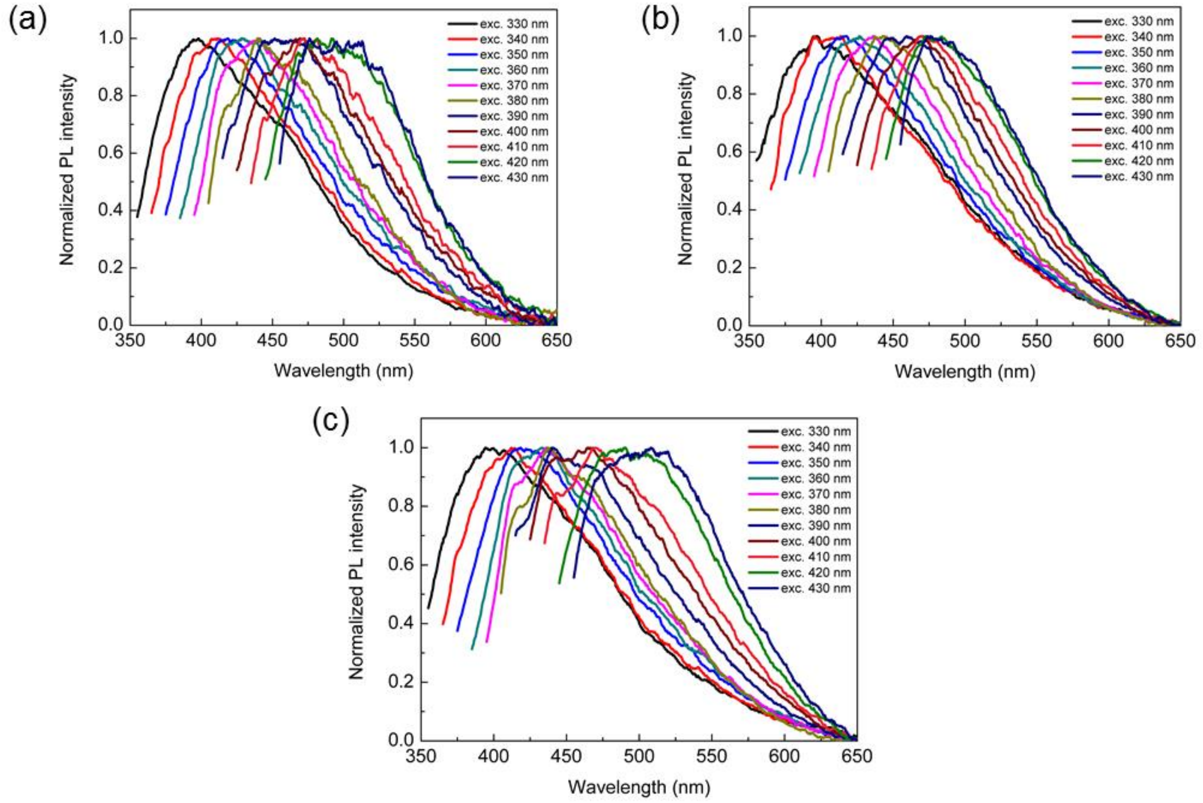

Figure S3: Normalized fluorescence spectra of peptide films H^+-F6-O^- (a), Ac-F6-O^- (b) and H^+-F6-Am (c) *versus* the excitation wavelength in the range between 330 and 430 nm

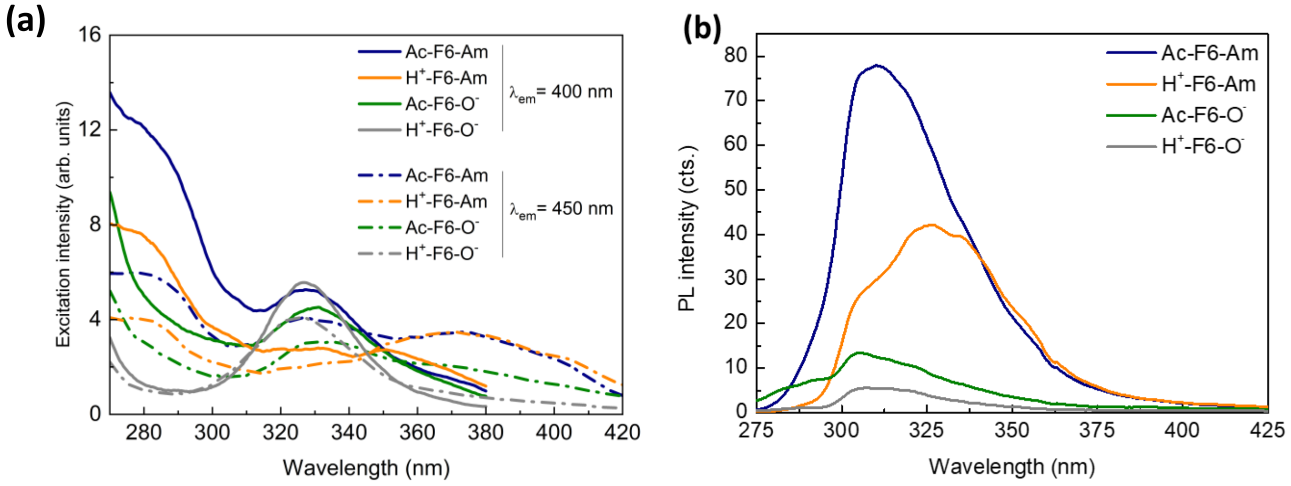

Figure S4: (a) Excitation spectra of the F6 setting λ_{em}= 400 nm (solid lines) and λ_{em} = 450 nm (dash-dotted lines) at 100 mg/mL concentration. (b) Fluorescence spectra of the F6 at λ_{exc} = 257 nm.

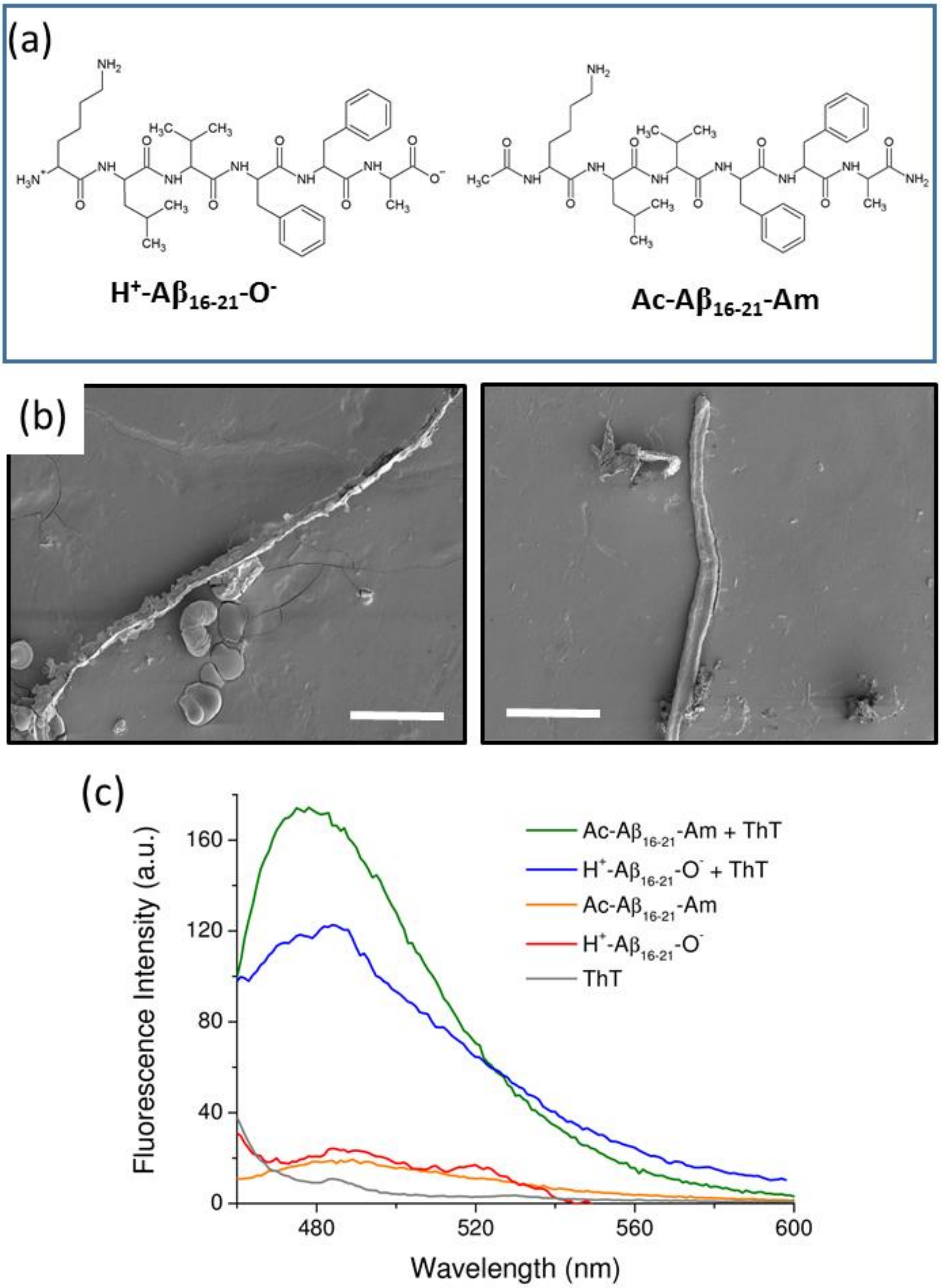

Figure S5: a) Schematic representation of the Aβ-peptide variants with charged/uncharged termini. b) Selected micrographs acquired on the Aβ-peptide solution at 5 mg/mL (H^+-A$\beta_{16\text{-}21}$-O^- on the left and Ac-A$\beta_{16\text{-}21}$-Am on the right) deposited on stub and air-dried (scale bar 100 μm). c) Fluorescence emission spectra of ThT, Aβ-peptides and the mix of ThT and peptides.
